# GLP-1 secretion in acute ischemic stroke: association with functional outcome and comparison with healthy individuals

**DOI:** 10.1186/s12933-019-0896-z

**Published:** 2019-07-15

**Authors:** Martin Larsson, Cesare Patrone, Mia von Euler, Jens J. Holst, David Nathanson

**Affiliations:** 1 Department of Clinical Science and Education at Södersjukhuset, Karolinska Institutet, Sjukhusbacken 10, 118 83 Stockholm, Sweden; 2 Department of Biomedical Sciences and NNF Center for Basic Metabolic Research, Faculty of Health and Medical Sciences, University of Copenhagen, Copenhagen, Denmark

**Keywords:** Glucagon like peptide-1, Ischemic stroke, Modified Rankin Scale (mRS), Hyperglycemia

## Abstract

**Background:**

Glucagon-like peptide-1 (GLP-1) treatment has been shown to reduce stroke incidence in diabetes and also to be neuroprotective in experimental stroke models. The prognostic value of endogenous levels of GLP-1 in the recovery phase after stroke remains to be elucidated. The aim of the study was to investigate the potential association between GLP-1 levels and functional outcome after stroke and to determine whether GLP-1 is altered in the acute phase of stroke compared to 3 months post stroke and to healthy controls.

**Methods:**

Fasting GLP-1 was measured on hospital day 2–4 in patients without previously known diabetes (n = 59) that received recombinant tissue plasminogen activator (rtPA) for ischemic stroke. Fasting GLP-1 was measured again after 3 months and neurologic outcome was measured as modified Rankin Scale (mRS). mRS ≥ 2 was considered as unfavorable outcome. A control group of healthy individuals (n = 27) was recruited and their fasting GLP-1 was measured.

**Results:**

Fasting GLP-1 was higher in the patients that suffered a stroke compared to healthy controls (25.1 vs. 18.0 pmol/L; p = 0.004). The GLP-1 levels did not change significantly at the 3-month follow up OGTT (25.8 vs. 25.6; p = 0.80). There was no significant association between GLP-1 levels and unfavorable mRS (OR 1.03, 95% CI 0.95–1.12, p = 0.50).

**Conclusions:**

Endogenous GLP-1 levels in patients that recently suffered an ischemic stroke are higher than in healthy controls and remained unchanged at the 3 months follow-up, possibly indicating an elevation of the levels of GLP-1 already pre-stroke. However, no association between endogenous GLP-1 and functional outcome of stroke 3 months post stroke was found.

## Background

Stroke is the second largest cause of death worldwide and one of the leading causes of disability in adults [1]. 80% of all strokes are caused by an ischemic event [2]. Patients with diabetes have approximately a doubled risk of suffering from an ischemic stroke and the risk for recurrence is higher [3–5]. Furthermore, the stroke outcome has been shown to be worse compared to individuals without diabetes [3]. Additionally, diabetes patients are at an increased risk of progressive loss in functional status after stroke [6]. Reperfusion treatments available for ischemic stroke are recombinant tissue plasminogen activator (rtPA) and, in selected cases, thrombectomy [7]. Treatment with rtPA carries a risk of hemorrhagic transformation, which is increased in hyperglycemic states [8]. Therefore, identification of new treatment strategies in diabetic patients suffering from stroke is highly needed.

Glucagon like peptide-1 (GLP-1) is a peptide hormone that is released from endocrine epithelial cells of the small intestine in response to a meal and is responsible for potentiating postprandial release of insulin, resulting in greater and more rapid insulin secretion [9, 10]. Receptors for GLP-1 are present in various cell types such as pancreatic, endothelial and myocardial cells. Additionally, GLP-1 receptors have been found in neurons, and in the brain [11].

Both experimental studies and large randomized trials have indicated that the treatment with stable GLP-1 analogues, both before and after onset of ischemic stroke, may be neuroprotective and may reduce the risk of ischemic stroke [12–18]. Protection from stroke by GLP-1 analogues was also recently shown in a meta-analysis by Barkas [19]. However, human studies evaluating effects of both exogenous and endogenous GLP-1 on functional outcome after stroke are utterly scarce and highly needed [20]. Moreover, previous data on the relationship between endogenous GLP-1 levels and cardiovascular disease are ambiguous [21, 22].

This study had three aims. Firstly to determine whether GLP-1 secretion is different in subjects at high risk for ischemic stroke compared to healthy controls. Secondly we wanted to determine if GLP-1 secretion is altered in the acute phase of ischemic stroke. The third aim was to evaluate if the GLP-1 level in the acute phase following ischemic stroke is associated with functional outcome 3 months post stroke.

## Methods

### Study population

All patients treated with rtPA for ischemic stroke at the hospital Södersjukhuset in Stockholm, Sweden, are included in a clinical quality of care registry. Among these patients, a research nurse consecutively screened patients. Consenting patients over 18 years that had been treated with rtPA for an ischemic stroke with preserved swallowing function and without a recent stroke (within 3 months) or previously known diabetes were eligible for participation. A control group of healthy volunteers between 18 and 80 years of age without previously known diabetes or vascular disease was recruited by advertisement. Use of the clinical quality of care registry also ensured access to baseline characteristics of the total cohort of rtPA treated patients during the study period. The Ethics Committee at Karolinska Institutet, Stockholm, approved the study and clinical register (No. 2012/626-31/4).

### OGTT

**Fig. 1:**
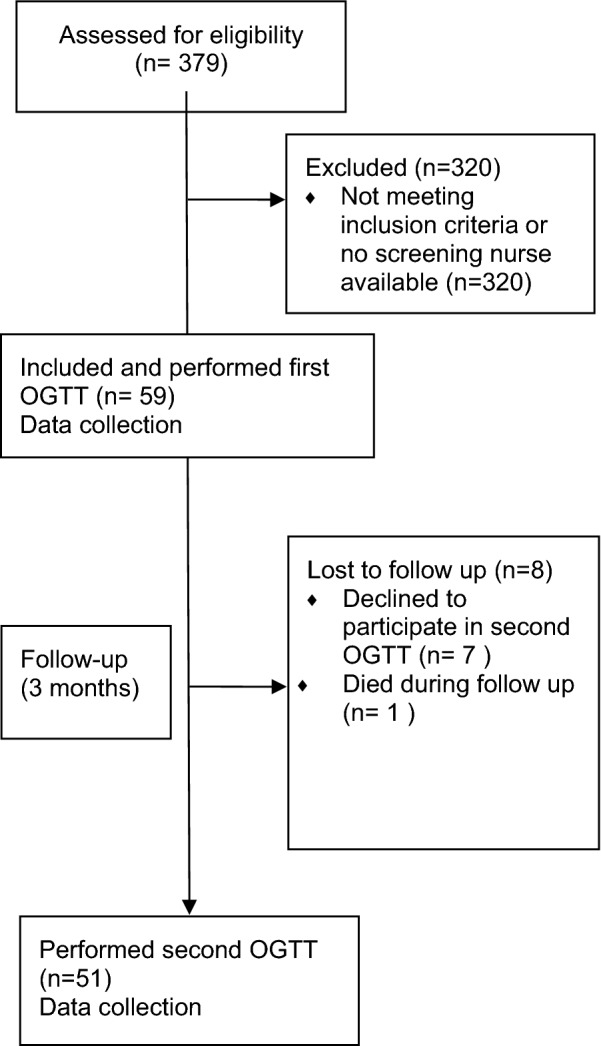
Overview of study events of the stroke patients

For blood sample sampling a 75 g Oral Glucose Tolerance Test (OGTT) was performed on the included patients at hospital day 2–4 of admission to hospital after overnight fasting. Stroke patients were followed up after 3 months with a second OGTT. Venous blood was sampled at 0 and 120 min. See Fig. 1. Glucose was measured as plasma glucose by the HemoCue 201+ system (HemoCue AB, Ängelholm, Sweden). The healthy controls underwent OGTT at one occasion. Plasma was separated immediately by centrifugation and frozen at − 70 °C for later analysis.

### GLP-1 measurement

Fasting plasma GLP-1 was measured as total GLP-1 by radioimmunoassay using the antiserum 89,390 in batch analysis of frozen plasma samples. This antibody detects both the 7-36 amide (intact GLP-1) and 9-36 amide (primary metabolite) forms of GLP-1 [23].

### Assessment of insulin sensitivity

Increased insulin resistance is a well-known predictor for cardiovascular disease [24–26]. The Cederholm Insulin Resistance Index (Ceder-IR) is a stronger predictor for incident cardiovascular disease compared to other insulin resistance indices (e.g. HOMA-IR [27, 28]. Ceder-IR was therefore used as a marker for insulin resistance in the present study. Ceder-IR is calculated during a 75 g OGTT by combining glucose and insulin levels at 0 and 120 min respectively together with body weight as described in full detail by Zethelius and Cederholm [28]. Plasma insulin was measured by electrochemiluminescence by the Karolinska University Laboratory in Stockholm, Sweden.

### Neurologic deficit and outcome measures

The National Institutes of Health Stroke Scale (NIHSS) [29] was used to estimate the severity of stroke and modified Rankin Scale (mRS) [30] to evaluate the outcome at 3 months after stroke. The baseline values were recorded together with risk factors, vital signs, cholesterol level, estimated glomerular filtration rate (eGFR) and anthropometric data at time of admission to the hospital. A trained research nurse determined mRS at follow-up after 3 months by face-to-face visit at time of the second OGTT or by telephone interview by investigator (ML) (n = 3). Functional outcome was classified as favorable if the mRS at 3 months was 0–1 and unfavorable if mRS was 2–6 [31].

### Statistical analyses

To compare fasting plasma GLP-1, fasting plasma insulin and plasma glucose levels during a 75 g OGTT between stroke patients and healthy controls, a Univariate General Linear Model (UNIANOVA) was used. Case or control status and time point during the OGTT was used as fixed factors. Adjustment was made for sex, age and BMI. An interaction term between time point during OGTT and case/control status was used.

To compare the fasting plasma GLP-1 and fasting plasma insulin between the acute phase and recovery phase after 3 months among the stroke patients Wilcoxon Signed Ranks Test was used. To compare the plasma glucose levels during the 75 g OGTT a 2-way ANOVA was used without adjustments (OGTT performed on same individuals at both times). To correct for sphericity violation Huynh–Feldt correction was applied.

To determine if fasting plasma GLP-1 in the acute phase could predict unfavorable outcome of stroke, Logistic Regression was used. In our final model, adjustment was made for age, sex, initial NIHSS, Ceder-IR and presence of atrial fibrillation. Hypertension, smoking status, previous stroke and renal function were tested as potential confounders but were excluded in the final model as they did not affect the OR significantly.

The only grouping of a variable was unfavorable outcome (mRS 2–6). For one individual in the control group one GLP-1 measurement was missing and because ANOVA requires full data sets that individual was excluded. No other data in the variables included in the models were missing.

A power calculation in preparation for the study indicated that a sample size of 46 patient treated with rtPA and 23 in the control group would give a power of 90% to detect a difference of 5 pmol/l in GLP-1 levels (22 vs. 17) between the groups.

SPSS v23.0.0.3 (IBM Corp, Armonk, NY, USA) was used for statistical analysis. A two sided p-value < 0.05 was considered statistically significant.

## Results

Of the 379 patients treated with rtPA at the hospital Södersjukhuset from January 2014 to January 2017, 59 patients were included in the present study. 51 completed double OGTT, 1 died during the follow up period and 7 declined a second OGTT. See Fig. 1. A control group of healthy volunteers was recruited by advertisement (n = 27). Baseline characteristics are presented in Table 1. Data is shown as median with interquartile range or number (n) with proportion where appropriate. For comparison, data regarding the total screened cohort (n = 379) is also included in Table 1. The stroke patients who underwent OGTT were older (70,6 vs. 44,6), comprised more males (66% vs. 41%) and had higher BMI (26 vs. 23) compared to the healthy controls.

**Table 1:** Baseline characteristics of the study population

Characteristic	OGTT in stroke (*n *= 59)	OGTT controls (*n *= 27)	Total rtPA cohort (*n *= 379)
Age, years	70.6 (61.5–76.2)	44.6 (27.9–68.3)	74.5 (65.3–83.3)
Male sex (%)	66.1	40.7	51.4
NIH before tPA	4 (3–6)	–	6 (3–12.5)
mRS discharge	1 (1–3)	–	2 (1–4)
BMI, kg/m^2^	26.2 (23.4–29.3)	22.7 (21.2–26.0)	24.9 (22.9–28.4)
eGFR, ml min^−1^ (1.73 m)^−2^	70 (59–76)	–	64 (51–76)
Admission glucose mmol/L	6.8 (6.0–7.5)	–	6.7 (5.9–8.0)
Hypertension	54%	–	56%
Systolic BP, mmHg	154 (140–176)	–	157 (140–173)
Diastolic BP, mmHg	89 (80–98.5)	–	83.5 (75–93)
Atrial fibrillation (%)	14	–	20
Current smokers (%)	10	–	12
Prev. diabetes (%)	0	0	15
Pathologic OGTT (%)	57.6	11.1	–
Normal glucose tolerance	42.4	88.9	–
Fasting hyperglycemia	18.6	3.7	–
Impaired glucose tolerance	52.5	11.1	–
Diabetes	5.1	0	–

Values are medians (IQR) or %

### GLP-1 levels are higher in patients that recently suffered ischemic stroke

**Fig. 2:**
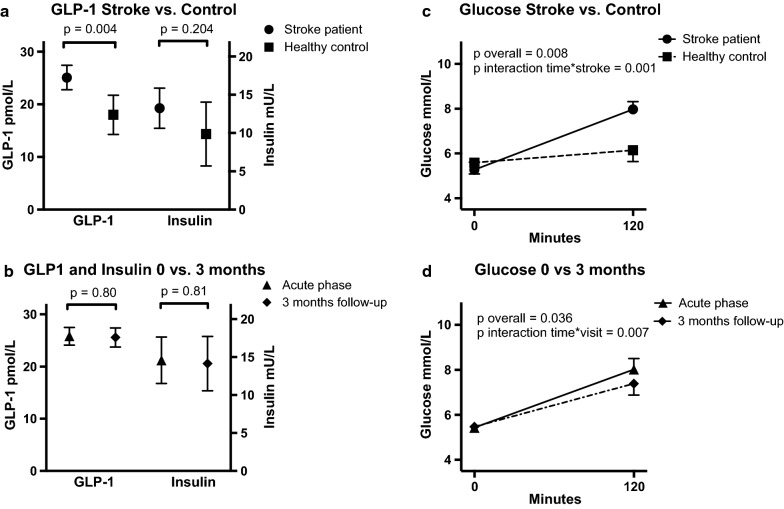
Fasting Plasma GLP-1 and fasting plasma insulin (**a**, **b**) and plasma glucose (**c**, **d**) during a 120 min OGTT. Data is presented as mean values, Bars indicate 95% CI. For **a**, **c** the graphs show means adjusted for sex, age and BMI

The levels of fasting GLP-1 in patients that recently suffered an ischemic stroke were elevated compared to healthy controls (25.1 vs. 18.0 pmol/L; p = 0.004), with a difference of 7.1 (95% CI 2.4–11.8). There was no evidence of difference in fasting plasma insulin (13.2 vs. 9.8; p = 0.20). The results are shown in Fig. 2a.

There was no significant difference between the fasting GLP-1 levels during the acute phase of the ischemic stroke and at the 3 months follow-up (25.8 vs. 25.6, p = 0.80). Nor was there any evidence of a difference in the insulin levels between the acute phase and the 3 months follow-up (14.7 vs. 14.1, p = 0.81) (Fig. 2b).

The glucose challenge (OGTT) demonstrated increased plasma glucose levels for the stroke patients compared to the healthy controls (7.8 vs. 6.1, p < 0.001), but there was no difference in their fasting glucose levels (5.3 vs. 5.6, p = 0.33) (Fig. 2c). The glucose levels during the acute stroke phase were higher during the OGTT when compared to the 3-month follow-up (8.0 vs. 7.4, p = 0.015). Displayed in Fig. 2d.

### No association between GLP-1 and stroke outcome

Fasting GLP-1 sampled at day 2–4 after an ischemic stroke in patients that were treated with rtPA was not significantly associated to unfavorable outcome. Unadjusted Odds Ratio (OR) was estimated to 1.03 (95% CI 0.95–1.12, p = 0.50). The results did not change after adjustments for sex, age and NIHSS, OR for unfavorable outcome was found to be 0.99 (95% CI 0.89–1.10, p = 0.85).

### Insulin sensitivity associated with stroke outcome

Ceder-IR was significantly associated to unfavorable outcome (mRS ≥ 2) at 3 months with OR of 1.7 (95% CI 1.2–2.6 p = 0.004).

## Discussion

### Principal findings

This study aimed at describing the secretion of GLP-1 in acute stroke and to determine if endogenous levels of this peptide was associated with functional outcome after rtPA treated ischemic stroke. Our results shows that plasma GLP-1 in patients who recently suffered an ischemic stroke and were treated with rtPA is higher compared to healthy controls. In the group with ischemic stroke GLP-1 levels remained unchanged 3 months after the stroke event. This finding could suggest that the elevation of GLP-1 was present before the stroke. However, the quite short follow up of 3 months does not rule out that the increased levels of GLP-1 in stroke patients could be an effect of the stroke itself and cautious interpretation is therefore warranted. Importantly, the endogenous GLP-1 levels were not associated with functional outcome of the stroke.

### Effects of GLP-1 on the cardiovascular system

GLP-1 treatment has been shown to reduce stroke incidence [16, 19] and in preclinical experimental studies GLP-1 based therapy has been associated with reduced stroke volume, improved neuronal survival and reduced neurologic deficit [12–15]. There are, however, no studies examining the endogenous levels of GLP-1 in patients that recently suffered a stroke and if the endogenous levels could predict the outcome of a stroke.

Clinical studies have shown preventive effects of GLP-1 analogue treatment on cardiovascular mortality and direct stimulation of the GLP-1R with the GLP-1 analogue liraglutide has also shown positive effect on cardiovascular parameters such as arterial stiffness [32, 33]. Interestingly inhibition of dipetidyl peptidase-4, the main enzyme responsible for inactivation of GLP-1, with sitagliptin resulting in higher levels of endogenous GLP-1 has shown positive effect on cardiac function in a diabetic setting [34]. Experimental animal studies have also shown that acute treatment with GLP-1 analogues and dipeptidyl peptidase-4 (DPP-4) inhibitors after a stroke is neuroprotective including better recovery [12, 35, 36], as recently reviewed [37–39]. Treatment with the GLP-1 analogue liraglutide after experimental stroke has been shown to promote angiogenesis and to improve functional outcome [18]. However, there are to date no clinical studies that have examined neuroprotective effects of neither endogenous nor pharmacologically administered GLP-1 in mitigating the impact of a stroke in humans [20].

The present work is to our knowledge the first to investigate the potential association between endogenous GLP-1 and stroke recovery, and the results indicate that endogenous fasting GLP-1 does not predict a favorable functional outcome after ischemic stroke.

### Strengths and weaknesses in relation to other studies

The ischemic stroke patients that underwent OGTT in our study were slightly younger than the total cohort of patients treated with rtPA for stroke during the study period. They also had milder strokes and the proportion of men was greater. These differences were expected, as women are older when they suffer strokes and the strokes are also worse compared to men [40]. The risk of swallowing impairment is also higher with larger strokes, which would make the patient ineligible for this study including an OGTT.

In comparison to the healthy control group, the group of stroke patients in our study was older, had a higher proportion of men and a higher BMI. Nevertheless, the differences in endogenous GLP-1 levels between the groups, with higher GLP-1 in stroke patients, remained significantly higher after adjustment for age, sex and BMI. Interestingly, a Danish study from 2015 showed that female sex is associated with higher GLP-1 levels during an OGTT [41]. Moreover, the study also reported a strong negative correlation between BMI and GLP-1 both for men and women [41]. This provides indirect evidence that the observed difference with higher GLP-1 among the stroke patients in our study represent a true association with stroke as our study population had more men and a higher BMI in the stroke group compared to the controls. Although speculative, these results could also suggest that GLP-1 might be elevated even before the stroke since increased GLP-1 plasma levels in stroke patients remained elevated 3 months after stroke. This could suggest a potential association between enhanced plasma GLP-1 and increased stroke risk. Future studies will have to confirm this hypothesis because there were no data available on the GLP-1 levels prior to the ischemic stroke. A few studies have looked into this with some discrepancies in the results. Higher fasting levels of GLP-1 have been reported in a different group of patients with vascular pathology such as aortic valve abnormality [42] suggesting that GLP-1 could be a broader marker for cardiovascular pathology. Other investigators have also found increased GLP-1 levels to be associated with higher risk for cardiovascular disease [21]. Notably, this correlation was independent of presence of diabetes. On the other hand, a study on patients with established coronary disease showed a negative correlation between GLP-1 and coronary disease [22]. Another possible explanation for our data with GLP-1 levels unchanged at 3 months after the stroke is that they are caused by the stroke with a lingering effect.

Previous studies have also shown that the GLP-1 response to an OGTT or meal is blunted in diabetes [43, 44]. It has also been confirmed in a large study that showed a 20% reduction in GLP-1 secretion in overweight and obese persons irrespective of glucose tolerance [41]. A smaller study in twins showed that in monozygotic pairs discordant for BMI and liver fat the GLP-1 secretion was reduced at 60 min during an OGTT [45]. Furthermore, an experimental study by Wang et al. has shown interesting results where 3-deoxyglucosone (3DG), a precursor for advanced glycation end-products known to be able to induce glucose intolerance, reduces GLP-1 release [46]. Against this background of GLP-1 level alterations our study finding with elevated GLP-1 levels in subjects with a recent ischemic stroke add information.

### Unanswered questions and future directions

A potential explanation for the fact that pharmacologically administered GLP-1 analogues show acute neuroprotective efficacy in the rodent while endogenous GLP-1 levels do not correlate with stroke recovery in patients could be that treatment with pharmacological agents in rodents are often given in higher doses. Conventional treatment in humans with exenatide also reaches concentrations about 2–3 times higher than endogenous GLP-1 as measured in our study [47]. Moreover, passage through the blood brain barrier has been shown for both GLP-1 and its analogues in the mouse [48–50]. However, whether and to what extent GLP-1 can pass the blood brain barrier in humans is undetermined. Differences in brain access could be responsible for differences in GLP-1-mediated neuroprotective efficacy. Other potential explanations for differential effects induced by endogenous GLP-1 and exogenous GLP-1 analogues could be explained by differential sets of cells responsible for such effects, as recently suggested by Vain et al. [51].

Previous studies have shown that the presence of stress, hyperglycemia or diabetes is detrimental to the outcome of ischemic stroke and is associated with higher degrees of hemorrhagic transformation [52, 53]. In our study we found a higher elevation of glucose in the acute phase after stroke, which reverted to more normal levels at the 3 months follow-up (Fig. 2). However, no significant alteration of the fasting secretion of GLP-1 during the follow-up was observed. Our finding that GLP-1 has no predictive value on the stroke-outcome argues against GLP-1 being an effective mediator for glycaemia regulation in patients exposed to an ischemic stroke and also suggest that GLP-1 is not part of the negative effect that hyperglycemia has on the outcome of stroke. All stroke patients in our study were treated with rtPA and an effect of this treatment on GLP-1 secretion is of course a possibility. This seems unlikely though because the levels remained unchanged at follow-up 3 months later. In addition, neither the stroke patient group nor the control group were treated with anti-diabetic drugs.

Insulin resistance has previously been reported to be a predictor of cardiovascular morbidity and mortality [24–26]. Therefore we adjusted for Ceder-IR as a potential confounder [28] which did not change the results significantly. Nevertheless, our data confirmed that insulin resistance is a predictor for unfavorable outcome measured by mRS. The fasting insulin levels are also shown in Fig. 2. Interestingly, we found no evidence of difference in insulin levels between the stroke patients and the control group. This finding must however be interpreted with caution as the study power was not calculated to detect differences in insulin levels and the lack of significant difference could be caused by larger variance in the insulin variable.

### Strengths and limitations of the study

A strength of this study is that the measurements were repeated on the same individuals, with a low number of patients lost to follow up (n = 8), giving the study a high internal validity. However, the exclusion of patients with known diabetes decreases the generalizability of the study. The relative small number of patients that underwent double OGTT (n = 51) may also be a limitation. The differences in age, sex, BMI and treatment between the control group and the stroke patients are also a weakness. Our regression model ought to have controlled for this differences. However, we cannot rule out the risk of residual confounding. Another limitation is that only fasting GLP-1 was measured in the present study. Since GLP-1 is a postprandial hormone it would have been interesting to also have the maximum stimulated level at 30 min post glucose load.

## Conclusion

Our study shows elevated GLP-1 levels in patients who recently suffered an ischemic stroke and were treated with rtPA with the levels being stable 3 months later, suggesting that this elevation could be a marker of an increased stroke risk but this would need confirmation in other trials with prospective design of longer follow-up. Moreover, the results must be interpreted with some caution as they could suggest an ongoing compensatory protective mechanism in individuals at high stroke risk. We also found that the plasma GLP-1 measured in the acute phase of stroke has no predictive value for the outcome of a stroke. Whether a more sustained increase of endogenous GLP-1 or administration of GLP-1 analogues in the recovery phase after stroke could improve neurological outcome and improve recovery are fundamental questions to be addressed in future studies.

## Data Availability

The dataset used and analyzed during the current study is available from the corresponding author on reasonable request.

## Abbreviations

3DG: 3-deoxyglucosone
Ceder-IR: Cederholm Insulin Resistance Index
DPP-4: dipeptidyl peptidase-4
GLP-1: glucagon-like peptide-1
HOMA-IR: homeostatic model assessment for insulin resistance
mRS: Modified Rankin Scale
NIHSS: National Institutes of Health Stroke Scale
rtPA: recombinant tissue plasminogen activator
